# 1872. Risk factors associated with gaps in screening and testing for latent TB infection in children

**DOI:** 10.1093/ofid/ofad500.1700

**Published:** 2023-11-27

**Authors:** Julia Fink, William Burrough, Charlotte Hsieh, Mariamawit Tamerat, Zarin Noor, Amit S Chitnis, Gena Lewis, Devan Jaganath

**Affiliations:** Lewis Katz School of Medicine, Temple University, Philadelphia, Pennsylvania; UCSF, San Francisco, California; UCSF, San Francisco, California; UCSF, San Francisco, California; UCSF, San Francisco, California; Alameda County Public Health Department, San Leandro, California; UCSF, San Francisco, California; University of California, San Francisco, San Francisco, California

## Abstract

**Background:**

Children should be treated for latent TB infection (LTBI) to prevent progression to TB disease, but there are gaps in screening and testing for LTBI. We evaluated demographic and clinical factors associated with LTBI screening and testing at a Federally Qualified Health Center in Oakland, California.

**Methods:**

We extracted demographic, clinical and note data from the electronic health records of all well-patient visits of children and adolescents 1-19 years old without a history of LTBI or TB disease from 2014 – 2020. Screening was defined as documenting the presence/absence of new TB risk factors, and testing was defined as ordering/completing a tuberculin skin test (TST) or QuantiFERON-TB Gold blood test (Quantiferon Gold). We performed multivariable logistic regression to determine the factors associated with conducting a TB risk assessment, ordering LTBI testing, and completing LTBI testing.

**Results:**

We evaluated 18,681 visits, of which 38% were age 1 – 4, 50% were female, and 19% preferred a language other than English. Overall, 90% of visits documented a TB risk assessment, of which 8% had a risk factor for TB infection. Only 26% visits with a documented TB risk had a TB test subsequently ordered. 82% of ordered tests were completed. We found that children 1 – 4 years old were more likely to be screened for TB risk factors than adolescents ≥12 years (aOR 7.87, 95% CI 6.77 – 9.13, p < 0.01), but adolescents were more likely to be tested (aOR 1.74, 95% CI 1.12-2.66, p < 0.05). Non-English-speaking patients were more likely to be assessed for TB risk factors and were more likely to have an LTBI test ordered (Table 1). LTBI testing was more likely to be completed in females (aOR 5.08, 95% CI 1.75 – 14.70, p < 0.01) as well as when TST was ordered compared to Quantiferon Gold (aOR 24.15, 95% CI 8.17 – 71.42, p < 0.01).

**Figure:**
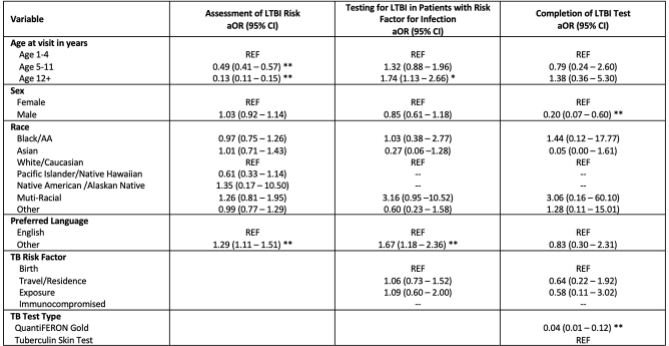

**Conclusion:**

Gaps in the LTBI care cascade for children were associated with age, sex and language preference, and interventions are needed to improve LTBI care in these groups. Further efforts are needed to address barriers to Quantiferon Gold completion in children.

**Disclosures:**

**All Authors**: No reported disclosures

